# Outcomes of Cardiovascular Magnetic Resonance Imaging in Patients Recently Recovered From Coronavirus Disease 2019 (COVID-19)

**DOI:** 10.1001/jamacardio.2020.3557

**Published:** 2020-07-27

**Authors:** Valentina O. Puntmann, M. Ludovica Carerj, Imke Wieters, Masia Fahim, Christophe Arendt, Jedrzej Hoffmann, Anastasia Shchendrygina, Felicitas Escher, Mariuca Vasa-Nicotera, Andreas M. Zeiher, Maria Vehreschild, Eike Nagel

**Affiliations:** 1 Institute for Experimental and Translational Cardiovascular Imaging, DZHK Centre for Cardiovascular Imaging, University Hospital Frankfurt, Frankfurt am Main, Germany; 2 Department of Biomedical Sciences and Morphological and Functional Imaging, University of Messina, Messina, Italy; 3 Department of Infectious Diseases, University Hospital Frankfurt, Frankfurt am Main, Germany; 4 Department of Diagnostic and Interventional Radiology, University Hospital Frankfurt, Frankfurt am Main, Germany; 5 Department of Cardiology, Goethe University Hospital Frankfurt, Frankfurt am Main, Germany; 6 Department of Hospital Therapy No. 1, I.M. Sechenov First Moscow State Medical University, Moscow, Russia; 7 Institute for Cardiac Diagnostic and Therapy, Berlin, Germany

## Abstract

**Importance:**

Coronavirus disease 2019 (COVID-19) continues to cause considerable morbidity and mortality worldwide. Case reports of hospitalized patients suggest that COVID-19 prominently affects the cardiovascular system, but the overall impact remains unknown.

**Objective:**

To evaluate the presence of myocardial injury in unselected patients recently recovered from COVID-19 illness.

**Design, Setting, and Participants:**

In this prospective observational cohort study, 100 patients recently recovered from COVID-19 illness were identified from the University Hospital Frankfurt COVID-19 Registry between April and June 2020.

**Exposure:**

Recent recovery from severe acute respiratory syndrome coronavirus 2 infection, as determined by reverse transcription–polymerase chain reaction on swab test of the upper respiratory tract.

**Main Outcomes and Measures:**

Demographic characteristics, cardiac blood markers, and cardiovascular magnetic resonance (CMR) imaging were obtained. Comparisons were made with age-matched and sex-matched control groups of healthy volunteers (n = 50) and risk factor–matched patients (n = 57).

**Results:**

Of the 100 included patients, 53 (53%) were male, and the mean (SD) age was 49 (14) years. The median (IQR) time interval between COVID-19 diagnosis and CMR was 71 (64-92) days. Of the 100 patients recently recovered from COVID-19, 67 (67%) recovered at home, while 33 (33%) required hospitalization. At the time of CMR, high-sensitivity troponin T (hsTnT) was detectable (greater than 3 pg/mL) in 71 patients recently recovered from COVID-19 (71%) and significantly elevated (greater than 13.9 pg/mL) in 5 patients (5%). Compared with healthy controls and risk factor–matched controls, patients recently recovered from COVID-19 had lower left ventricular ejection fraction, higher left ventricle volumes, and raised native T1 and T2. A total of 78 patients recently recovered from COVID-19 (78%) had abnormal CMR findings, including raised myocardial native T1 (n = 73), raised myocardial native T2 (n = 60), myocardial late gadolinium enhancement (n = 32), or pericardial enhancement (n = 22). There was a small but significant difference between patients who recovered at home vs in the hospital for native T1 mapping (median [IQR], 1119 [1092-1150] ms vs 1141 [1121-1175] ms; *P* = .008) and hsTnT (4.2 [3.0-5.9] pg/dL vs 6.3 [3.4-7.9] pg/dL; *P* = .002) but not for native T2 mapping. None of these measures were correlated with time from COVID-19 diagnosis (native T1: *r* = 0.07; *P* = .47; native T2: *r* = 0.14; *P* = .15; hsTnT: *r* = −0.07; *P* = .50). High-sensitivity troponin T was significantly correlated with native T1 mapping (*r* = 0.33; *P* < .001) and native T2 mapping (*r* = 0.18; *P* = .01). Endomyocardial biopsy in patients with severe findings revealed active lymphocytic inflammation. Native T1 and T2 were the measures with the best discriminatory ability to detect COVID-19–related myocardial pathology.

**Conclusions and Relevance:**

In this study of a cohort of German patients recently recovered from COVID-19 infection, CMR revealed cardiac involvement in 78 patients (78%) and ongoing myocardial inflammation in 60 patients (60%), independent of preexisting conditions, severity and overall course of the acute illness, and time from the original diagnosis. These findings indicate the need for ongoing investigation of the long-term cardiovascular consequences of COVID-19.

## Key Points

**Question:**

What are the cardiovascular effects in unselected patients with recent coronavirus disease 2019 (COVID-19)?

**Findings:**

In this cohort study including 100 patients recently recovered from COVID-19 identified from a COVID-19 test center, cardiac magnetic resonance imaging revealed cardiac involvement in 78 patients (78%) and ongoing myocardial inflammation in 60 patients (60%), which was independent of preexisting conditions, severity and overall course of the acute illness, and the time from the original diagnosis.

**Meaning:**

These findings indicate the need for ongoing investigation of the long-term cardiovascular consequences of COVID-19.

## Teaser

This cohort study evaluates the presence of myocardial injury in unselected patients recently recovered from coronavirus disease 2019 (COVID-19).

## Introduction

The global pandemic of coronavirus disease 2019 (COVID-19) continues to cause considerable morbidity and mortality worldwide.^1^ Thus far, the main emphasis of the research communication has been on acute respiratory complications, especially in critically ill patients. A number of case reports and small series suggested that COVID-19 prominently affects the cardiovascular system by exacerbating heart failure in patients with preexisting cardiac conditions^1,2,3^ and troponin elevation in critically ill patients.^4^ Fulminant myocarditis was suspected in 7% of patients with lethal outcome.^5^ The proposed pathophysiological mechanisms of cardiac injury include inflammatory plaque rupture, stent thrombosis, cardiac stress due to high cardiac output, and infection via the angiotensin-converting enzyme 2 receptors causing systemic endothelitis.^6,7^ A small number of autopsy cases suggest infiltration by interstitial mononuclear inflammatory cells,^8^ suggesting myocardial inflammation as the underlying mechanism, and some severe cases of myocarditis have been reported.^3,9^ In a small study of recovered patients with ongoing cardiac symptoms, cardiovascular magnetic resonance (CMR) imaging revealed cardiac involvement in 58% of patients consisting of myocardial edema and scar by late gadolinium enhancement (LGE).^10^ There remains poor insight into the cardiovascular sequelae in unselected patients, including those with no preexisting conditions, who were not hospitalized, or had no or only mild symptoms. To better understand the prevalence, extent, and type of cardiovascular sequelae, we proactively examined patients with a documented recent COVID-19 infection using serological markers of cardiac injury and highly standardized in-depth imaging with CMR.

## Methods

### Study Design and Participants

This is a prospective observational cohort study of 100 patients diagnosed with severe acute respiratory syndrome coronavirus 2 by reverse transcription–polymerase chain reaction on swab test of the upper respiratory tract who fulfilled inclusion criteria for this CMR investigation. This study followed the Strengthening the Reporting of Observational Studies in Epidemiology (STROBE) reporting guideline (eFigure in the Supplement). Participants were identified from the University Hospital Frankfurt COVID-19 Registry via the Department of Infectious Diseases and the Institute for Experimental and Translational Cardiovascular Imaging, Hesse, Germany, and were recruited between April and June 2020. All participants were considered eligible after a minimum of 2 weeks from the original diagnosis if they had resolution of respiratory symptoms and negative results on a swab test at the end of the isolation period. Patients recently recovered from COVID-19 referred for a clinical CMR due to active cardiac symptoms were not included in this analysis. Exclusion criteria were unwillingness to participate or provide informed consent or absolute contraindications for a contrast-enhanced magnetic resonance study. The study protocol was approved by the institutional ethics committee of the University Hospital Frankfurt (Improving Cardiovascular Risk Stratification Using T1 Mapping in General Population study^11^). Comparisons were made with age-matched and sex-matched control groups of normotensive adults who were taking no cardiac medications, had normal cardiac volumes and function, and had no evidence of scar (healthy controls; n = 50). Comparisons were also made with risk factor–matched patients (n = 57) for age, sex, hypertension, diabetes, smoking, known coronary artery disease, or comorbidities, sourced from the International T1 Multicenter Outcome Study.^12^ All procedures were performed in concordance with the Declaration of Helsinki and International Conference on Harmonization of Good Clinical Practice. All patients provided written informed consent.

Clinical demographic characteristics, medications, blood test results, endomyocardial biopsy results, and imaging measurements on the day of CMR examination were recorded using REDCap electronic data capture tools.^13^ All participants underwent venous blood sampling immediately prior to the CMR study. Blood samples were processed using standardized commercially available test kits for analysis of high-sensitivity troponin T (hsTnT) and N-terminal pro–b-type natriuretic peptide (Elecsys 2010; Roche). The local laboratory cutoff value for detectable hsTnT was greater than 3 pg/mL, whereas values above the 99th percentile (13.9 pg/mL) counted as a significant increase.^14^

### CMR Data Acquisition and Postprocessing

Cardiac magnetic resonance imaging was performed on clinical 3-T scanners (Magnetom Skyra; Siemens Healthineers), using standardized and unified imaging protocols (Goethe CVI Approaches). Conventional sequences were used for acquisition of cardiac function, volumes, mass, and scar imaging. Myocardial T1 and T2 mapping were acquired in a single midventricular short-axis slice using a validated variant of a modified Look-Locker Imaging sequence (Goethe CVI MOLLI), whereas for T2 mapping, a validated sequence for measurement of myocardial edema was used (T2-FLASH).^15,16,17^ Due to the proven sensitivity of Goethe CVI MOLLI for abnormal myocardium and evidence of superior diagnostic and prognostic performance,^18^ postcontrast T1 mapping was not part of the standardized protocol. Late gadolinium enhancement imaging was performed approximately 10 minutes after administration of 0.1 mmol/kg of body weight of gadobutrol (Gadovist; Bayer).

Cardiac volumes, function, and mass were measured using an artificial intelligence–based automated contour detection with manual correction if required (SuiteHeart; Neosoft). Myocardial T1 and T2 relaxation times were measured conservatively within the septal myocardium of the midventricular SAX slice using motion-corrected images, as per internal standardized operating procedures^19^ and with quality control by the core laboratory staff, blinded to the underlying clinical information using pseudonymized data sets. Areas of LGE were excluded from the measurements to avoid confounding diffuse fibrosis with replacement scar. Interpretation of LGE images followed standardized postprocessing recommendations; myocardial LGE was visually defined by 2 observers based on the presence and predominant pattern as ischemic or nonischemic.^20^ Pericardial LGE was considered present when enhancement involved both pericardial layers, irrespective of the presence of pericardial effusion. The distinction from the pericardial fat was ascertained using T1 mapping images.

### Statistical Analysis

Normality of distributions were tested using Shapiro-Wilk test. Categorical data are presented as counts (percentages) and continuous variables as means (standard deviation) and medians (interquartile ranges [IQRs]). Comparisons between patients’ groups were conducted using one-way analysis of variance for normally distributed parameters and Kruskal-Wallis for nonnormally distributed data with post hoc tests for significance between groups. Fischer exact and χ^2^ tests were used for proportions. Receiver operating characteristic curve analyses were used to examine discrimination (expressed as area under the receiver operating characteristic curve) between patients recently recovered from COVID-19 and control groups. Associations were explored using Pearson or Spearman correlation analyses, as appropriate for the type of data. Abnormal native T1 and T2 values were defined as greater than 1105 ms and greater than 37.4 ms, respectively, based on previously derived sequence-specific cutoffs of 2 SDs above the respective means in a healthy population.^18,21,22^ Significant abnormalities were defined as greater than 1136 ms for T1 and greater than 40 ms for T2, using 4 SDs above those means. Classification into abnormal and significantly abnormal served to distinguish the patients with a potential high risk of adverse events.^23,24^ All tests were 2-tailed, and *P* values less than .05 were considered statistically significant. Analysis was performed using SPSS software version 25.0 (IBM) and RStudio version 1.2.5001 (RStudio).

## Results

An unselected cohort of 100 patients who recently recovered from COVID-19 infection were included, of which 53 (53%) were male, and the mean (SD) age was 49 (14) years. Baseline characteristics are provided in Table 1. Most patients recovered at home (n = 67), with severity of the acute COVID-19 illness ranging from asymptomatic (n = 18) to minor to moderate symptoms (n = 49). A total of 33 severely unwell patients (33%) required hospitalization. In this group, 2 patients (2%) underwent mechanical ventilation, and 17 (17%) underwent noninvasive ventilation with positive airway pressure. Oxygen supplementation was required in 28 patients. In addition to respiratory support, patients received antiviral (n = 1), antibiotic (n = 15), and steroid (n = 8) therapy. Treatment with hydrochloroquine was initiated in a single patient but discontinued within days due to severe leukopenia. During hospitalization, a significant rise (greater than 13.9 pg/mL) in hsTnT values was documented in 15 patients (15%). Preexisting cardiovascular conditions included hypertension, diabetes, and known coronary artery disease but no previously known heart failure or cardiomyopathy. Other significant conditions included asthma (n = 10) and chronic obstructive pulmonary disease (n = 11). All preexisting conditions were similarly distributed between patients who recovered at home vs hospitalized.

**Table 1.** Patient Characteristics, Cardiac Magnetic Resonance (CMR) Imaging Findings, and Blood Test Results on the Day of CMR Examination

Characteristic	COVID-19 (n = 100)	Healthy controls (n = 50)	Risk factor–matched controls (n = 57)	*P* value^a^
**Patient characteristics**				
Age, mean (SD), y	49 (14)	48 (16)	49 (13)	.91
Male, No. (%)	53 (53)	25 (50)	28 (49)	.88
BMI, median (IQR)^b^	25 (23-28)	23 (20-25)^c^	27 (23-29)	<.001
Hypertension, No. (%)	22 (22)	0^c^	14 (25)	.003
Diabetes, No. (%)	18 (18)	0^c^	12 (22)	.002
Hypercholesterolemia, No. (%)	22 (22)	0^c^	13 (23)	.02
Known CAD, No. (%)	13 (13)	0^c^	9 (16)	.02
Smoking, No. (%)	22 (22)	9 (18)	11 (19)	.54
COPD or asthma, No. (%)	21 (21)	0^c^	13 (23)	.002
Blood pressure, mean (SD), mm Hg				
Systolic	129 (16)	122 (10)^c^	130 (15)	.006
Diastolic	80 (9)	75 (7)^c^	79 (12)	.03
Heart rate, mean (SD), beats per min	67 (10)	64 (10)	67 (12)	.17
SCORE, median (IQR), %	4 (2-6)	NA	4 (3-6)	.31
**CMR findings**				
LVEF, mean (SD), %	57 (6)	60 (5)^c^	62 (8)^c^	<.001
LVEDV index, mean (SD), mL/m^2^	86 (13)	80 (11)^c^	76 (14)^c^	<.001
LV mass index, mean (SD), g/m^2^	48 (9)	51 (12)	53 (12)^c^	.005
RVEF, mean (SD), %	54 (7)	60 (8)^c^	59 (9)^c^	<.001
Native T1, median (IQR), ms	1125 (1099-1157)	1082 (1067-1097)^c^	1111 (1098-1124)^c^	<.001
Abnormal native T1, No. (%)	73 (73)	6 (12)^c^	33 (58)^c^	<.001
Significantly abnormal native T1, No. (%)	40 (40)	0^c^	7 (12)^c^	<.001
Native T2, mean (SD), ms	38.2 (2.0)	35.7 (1.5)^c^	36.4 (1.6)^c^	<.001
Abnormal native T2, No. (%)	60 (60)	6 (12)^c^	15 (26)^c^	<.001
Significantly abnormal native T2, No. (%)	22 (22)	0^c^	0^c^	<.001
LGE, No. (%)				
Myocardial	32 (32)	0^c^	9 (17)^c^	<.001
Nonischemic	20 (20)	0^c^	4 (7)^c^	<.001
Pericardial	22 (22)	0^c^	8 (14)	<.001
Pericardial effusion (>10 mm), No. (%)	20 (20)	0^c^	4 (7)^c^	<.001
**Blood test results**				
High-sensitivity CRP, median (IQR), mg/dL	0.11 (0.06-0.20)	0.11 (0.04-0.19)^c^	0.07 (0.04-0.14)^c^	.007
hsTnT, median (IQR), pg/mL	4.9 (3.0-6.9)	3.0 (3.0-3.0)^c^	3.2 (3.0-4.5)^c^	<.001
Detectable hsTnT (>3 pg/mL), No. (%)	71 (71)	11 (22)^c^	31 (54)^c^	<.001
Significantly elevated hsTnT (>13.9 pg/mL), No. (%)	5 (5)	0	0	.06
NT-proBNP, median (IQR), pg/mL	51 (31-96)	47 (32-63)	59 (35-76)	.26

Abbreviations: BMI, body mass index; CAD, coronary artery disease; COPD, chronic obstructive pulmonary disease; COVID-19, coronavirus disease 2019; CRP, C-reactive protein; hsTnT, high-sensitivity troponin T; IQR, interquartile range; LGE, late gadolinium enhancement; LV, left ventricle; LVEDV, left ventricular end-diastolic volume; LVEF, left ventricular ejection fraction; NA, not applicable; NT-proBNP, N-terminal pro–b-type natriuretic peptide; RVEF, right ventricular ejection fraction; SCORE, Systematic Coronary Risk Evaluation.

^a^ *P* value for 3-group comparison using one-way analysis of variance or Kruskall Wallis, as appropriate for the type of data.

^b^ Calculated as weight in kilograms divided by height in meters squared.

^c^ Post hoc test for the difference vs COVID-19 group, *P* < .05.

**Figure 1.**
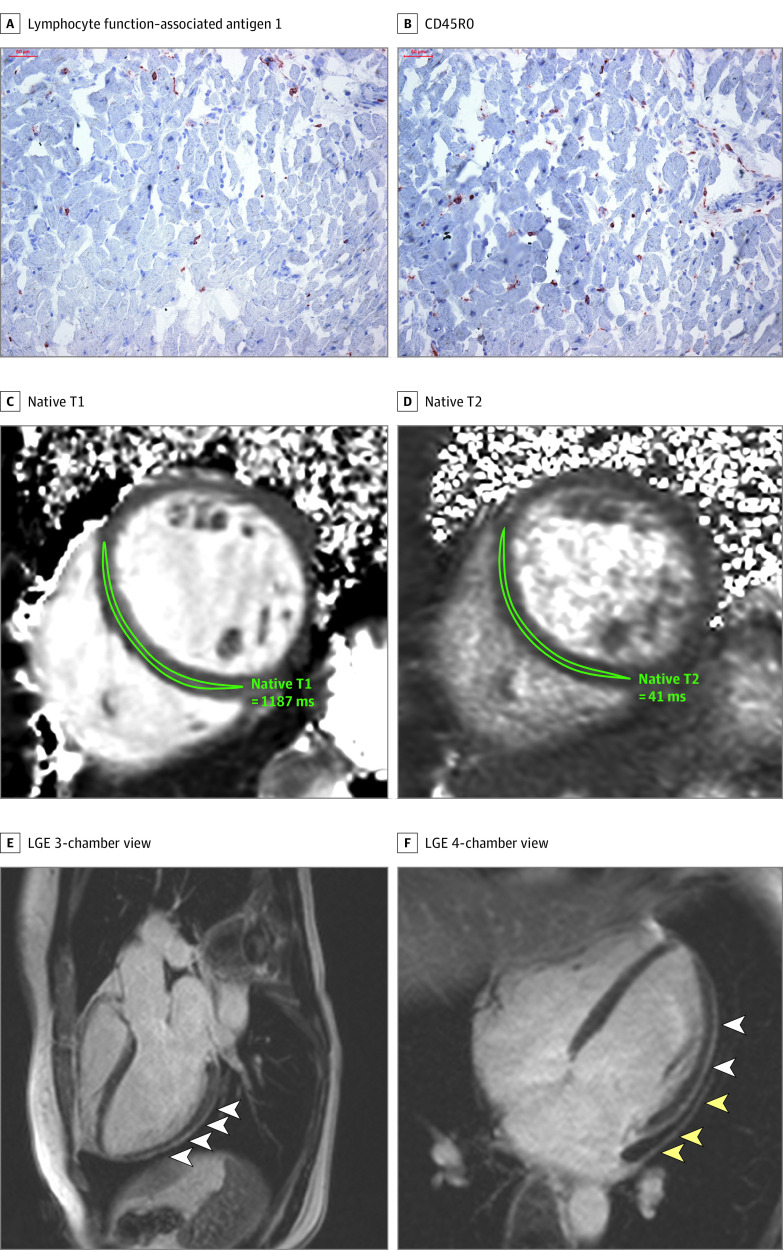
Representative Histologic and Cardiac Magnetic Resonance Imaging Abnormalities in 2 Patients After Coronavirus Disease 2019 (COVID-19) Diagnosis A and B, Histologic findings in an adult man with severe cardiac magnetic resonance imaging abnormalities 67 days after COVID-19 diagnosis. High-sensitivity troponin T level on the day of cardiac magnetic resonance imaging was 16.7 pg/mL. The patient recovered at home from COVID-19 illness with minimal symptoms, which included loss of smell and taste and only mildly increased temperature lasting 2 days. There were no known previous conditions or regular medication use. Histology revealed intracellular edema as enlarged cardiomyocytes with no evidence of interstitial or replacement fibrosis. Panels A and B show immunohistochemical staining, which revealed acute lymphocytic infiltration (lymphocyte function–associated antigen 1 and activated lymphocyte T antigen CD45R0) as well as activated intercellular adhesion molecule 1. C to F, Representative cardiac magnetic resonance images of an adult woman with COVID-19–related perimyocarditis. Panels C and D show significantly raised native T1 and native T2 in myocardial mapping acquisitions. Panels E and F show pericardial effusion and enhancement (yellow arrowheads) and epicardial and intramyocardial enhancement (white arrowheads) in late gadolinium enhancement (LGE) acquisition.

**Figure 2.**
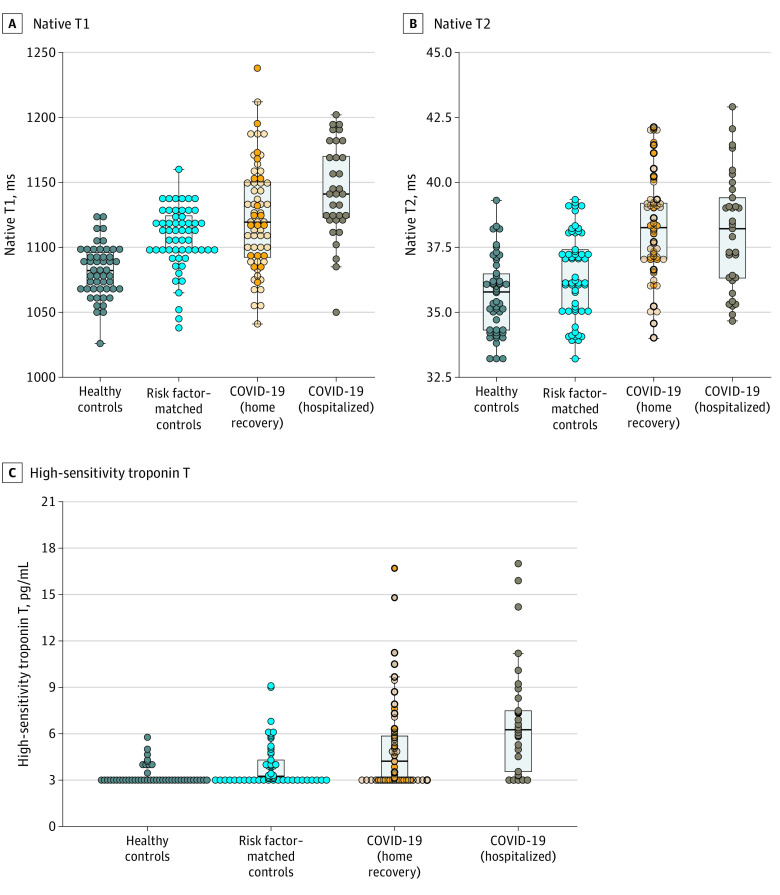
Scatterplots of Native T1, Native T2, and High-Sensitivity Troponin T Measures by Group There was a small but significant difference between patients who recovered at home vs in the hospital for native T1 (median [interquartile range], 1119 [1092-1150] ms vs 1141 [1121-1175] ms; *P* = .008) and high-sensitivity troponin T (4.2 [3.0-5.9] pg/dL vs 6.3 [3.4-7.9] pg/dL; *P* = .002) but not for native T2 or N-terminal pro–b-type natriuretic peptide. For the coronavirus disease 2019 (COVID-19) home recovery group, dark circles indicate symptomatic illness and light circles indicate asymptomatic illness. Boxes indicate overlays of box-whisker plots, midlines indicate medians, and whiskers indicate the farthest data point not regarded as an outlier (ie, within 1.5-fold the interquartile range).

**Figure 3.**
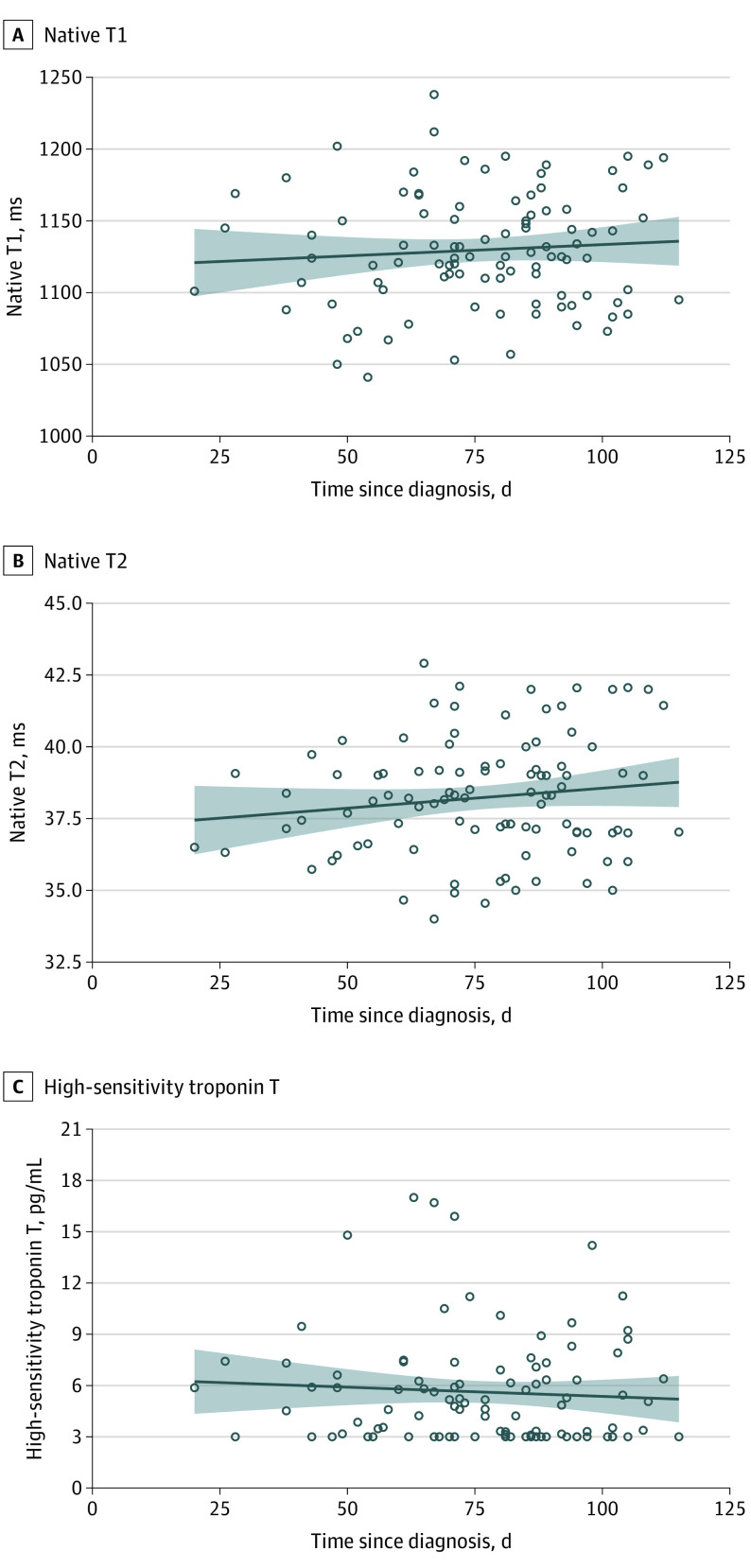
Correlation of Myocardial Measures With Time From Coronavirus Disease 2019 (COVID-19) Testing There was no significant correlation with duration between the positive test for COVID-19 and the measures (native T1: *r* = 0.07; *P* = .47; native T2: *r* = 0.14; *P* = .15; high-sensitivity troponin T: *r* = −0.07; *P* = .50). The trend line indicates the linear regression trend, and the shaded area indicates 95% CIs of the mean.

Patient characteristics and the results of the imaging parameters and blood markers on the day of CMR are shown in Table 1. Body mass index, hypertension, diabetes, hypercholesterolemia, known coronary artery disease, and chronic obstructive pulmonary disease or asthma were associated with COVID-19 diagnosis compared with the healthy controls, but there were no differences between those with COVID-19 and the risk factor–matched patients. The median (IQR) duration between the positive COVID-19 testing and the CMR examination was 71 (64-92) days. On the day of CMR examination, direct questioning about symptoms revealed atypical chest pain (n = 17) and palpitations (n = 20). Compared with pre–COVID-19 status, 36 patients (36%) reported ongoing shortness of breath and general exhaustion, of whom 25 noted symptoms during less-than-ordinary daily activities, such as a household chore. Only 4 of these 25 patients (16%) were previously hospitalized. No patient reported typical angina symptoms or a recent syncope. High-sensitivity troponin T values were detectable (greater than 3 pg/mL) in 71 patients recently recovered from COVID-19 (71%) and significantly elevated (greater than 13.9 pg/mL) in 5 (5%). Compared with healthy controls and risk factor–matched controls, patients recently recovered from COVID-19 had lower left ventricular and right ventricular ejection fraction, higher left ventricular volume, and raised native T1 and T2 measures. A total of 78 patients recently recovered from COVID-19 had abnormal CMR findings, including at least one of the following: raised myocardial native T1 (n = 73),^21^ raised myocardial native T2 (n = 60),^22^ myocardial LGE (n = 32), or pericardial enhancement (n = 22) (Figure 1). A total of 12 patients recently recovered from COVID-19 had an ischemic-type pattern of myocardial LGE. Three patients with severe abnormalities (significantly higher hsTnT, native T1, and native T2 measures, LGE, and left ventricular ejection fraction less than 50%) were referred to endomyocardial biopsy, revealing active lymphocytic inflammation with no evidence of any viral genome. Figure 2 and Figure 3 show the findings for native T1 and T2 mapping and hsTnT values based on the COVID-19 illness presentation (home-based recovery vs hospitalization) and in relation to the time from the original COVID-19 diagnosis. There was a significant difference between patients who recovered at home vs in the hospital for native T1 measures (median [IQR], 1119 [1092-1150] ms vs 1141 [1121-1175] ms; *P* = .008) and hsTnT (4.2 [3.0-5.9] pg/dL vs 6.3 [3.4-7.9] pg/dL; *P* = .002) but not for native T2 or N-terminal pro–b-type natriuretic peptide. There was no significant correlation with duration between the positive test for COVID-19 and the measures (native T1: *r* = 0.07; *P* = .47; native T2: *r* = 0.14; *P* = .15; hsTnT: *r* = −0.07; *P* = .50) (Figure 3). High-sensitivity troponin T was significantly correlated with native T1 (*r* = 0.33; *P* < .001), native T2 (*r* = 0.18; *P* = .01), and left ventricle mass (*r* = 0.25; *P* = .01). There was also a cross-correlation between native T1 and T2 (*r* = 0.40; *P* < .001). The associations of hsTnT with mapping measures remained significant despite controlling for the presence of comorbidities (overall or separately) or treatment received during the COVID-19 illness.

Table 2 shows the results of the receiver operating characteristic curve analyses for discrimination between the control groups and patients recently recovered from COVID-19 using imaging measures and blood biomarkers. Native T1 and T2 were the measures with the best discriminatory ability to detect COVID-19–related myocardial pathology.

**Table 2.** Results of the Receiver Operating Characteristic Curve Analyses

Characteristic	COVID-19 vs healthy controls		COVID-19 vs risk factor–matched controls	
	AUC (95% CI)	*P* value	AUC (95% CI)	*P* value
Native T1	0.86 (0.80-0.92)	<.001	0.76 (0.69-0.83)	<.001
Native T2	0.84 (0.78-0.91)	<.001	0.79 (0.73-0.85)	<.001
LVEF	0.70 (0.62-0.79)	<.001	0.62 (0.52-0.71)	.01
LVEDV index	0.65 (0.56-0.73)	.001	0.67 (0.58-0.75)	<.001
LV mass index	0.60 (0.49-0.70)	.07	0.63 (0.54-0.71)	<.001
RVEF	0.74 (0.66-0.83)	.001	0.61 (0.52-0.70)	.02
hsTnT	0.79 (0.72-0.86)	<.001	0.72 (0.57-0.76)	<.001
NT-proBNP	0.56 (0.46-0.65)	.21	0.52 (0.44-0.61)	.58
High-sensitivity CRP	0.54 (0.44-0.64)	.48	0.60 (0.52-0.76)	.05

Abbreviations: AUC, area under the receiver operating characteristic curve; COVID-19, coronavirus disease 2019; CRP, C-reactive protein; hsTnT, high-sensitivity troponin T; LV, left ventricle; LVEDV, left ventricular end-diastolic volume; LVEF, left ventricular ejection fraction; NT-proBNP, N-terminal pro–b-type natriuretic peptide; RVEF, right ventricular ejection fraction.

## Discussion

A total of 78 patients who recovered from COVID-19 infection (78%) had cardiovascular involvement as detected by standardized CMR, irrespective of preexisting conditions, the severity and overall course of the COVID-19 presentation, the time from the original diagnosis, or the presence of cardiac symptoms. The most prevalent abnormality was myocardial inflammation (defined as abnormal native T1 and T2 measures), detected in 60 patients recently recovered from COVID-19 (60%), followed by regional scar and pericardial enhancement. Findings on classic parameters, such as volumes and ejection fractions, were mildly abnormal. Myocardial measures, native T1 measures, and native T2 measures provided the best discriminatory value against healthy controls and risk factor–matched controls for exclusion of any myocardial disease or confirmation of COVID-19–related involvement, respectively.

To our knowledge, this is the first prospective report on a cohort of unselected patients with a recent COVID-19 infection identified from a local testing center who voluntarily underwent evaluation for cardiac involvement with CMR. The results of our study provide important insights into the prevalence of cardiovascular involvement in the early convalescent stage. Our findings demonstrate that participants with a relative paucity of preexisting cardiovascular condition and with mostly home-based recovery had frequent cardiac inflammatory involvement, which was similar to the hospitalized subgroup with regards to severity and extent. Our observations are concordant with early case reports in hospitalized patients showing a frequent presence of LGE,^3,25^ diffuse inflammatory involvement,^10,26^ and significant rise of troponin T levels.^4^ Unlike these previous studies, our findings reveal that significant cardiac involvement occurs independently of the severity of original presentation and persists beyond the period of acute presentation, with no significant trend toward reduction of imaging or serological findings during the recovery period. Our findings may provide an indication of potentially considerable burden of inflammatory disease in large and growing parts of the population and urgently require confirmation in a larger cohort. Although the long-term health effects of these findings cannot yet be determined, several of the abnormalities described have been previously related to worse outcome in inflammatory cardiomyopathies.^27,28,29^ Most imaging findings point toward ongoing perimyocarditis after COVID-19 infection. This is further confirmed by the cross-correlation between the T1 and T2 measures and hsTnT as well as histological verification of inflammatory changes in more severe cases.

Each of the abnormal imaging parameters can be linked to an underlying pathophysiological process and worse outcome. The peri-epicardial LGE in the areas with increased contrast agent uptake represents regional damage due to myocardial inflammation. Especially in combination with pericardial effusion, these observations can be attributed to fibrosis and/or edema due to an ongoing active pericarditis. Nonischemic patterns of myocardial LGE are mainly observed in patients with acute or healed myocarditis and have been strongly linked to reduced outcome.^23,24,30,31^ Increased native T1 measures represent diffuse myocardial fibrosis and/or edema, whereas native T2 is specific for edema.^18^ Thus, patients with increased native T1 and T2 measures have an active inflammatory process, while those with increased native T1 and normal native T2 measures have healed with some residual diffuse myocardial damage (although native T1 measures can be increased in a variety of pathophysiology, as many different pathways lead to diffuse fibrosis, including hypertension or genetic cardiomyopathies). Yet the combination with histological findings as well as the increase relative to age-matched, sex-matched, and risk factor–matched controls makes a COVID-19–related inflammatory process as the underlying pathophysiology highly likely. Increased native T1 measures have been strongly related to worse outcome in patients with ischemic heart disease and nonischemic cardiomyopathies.^23,24,30,31^ Increased troponin T and C-reactive protein levels similarly indicate inflammatory and partially ongoing myocardial damage and have been related to worse outcome, even if only minimally increased.^32^ While left and right ventricular ejection fraction were significantly reduced, there was a large overlap between patients recently recovered from COVID-19 and both control groups, demonstrating that volumes and function are inferior markers of disease detection compared with direct tissue characterization with mapping measures. Importantly, volumes and function have consistently been demonstrated to be less relevant for predicting outcome than LGE and mapping, highlighting the relevance of the more sensitive markers of early cardiac damage.^23,24,30,31^

### Limitations

Our study has limitations. The findings are not validated for the use in pediatric patients 18 years and younger. They also do not represent patients during acute COVID-19 infection or those who are completely asymptomatic with COVID-19. Several patients within our cohort had new or persistent symptoms, thus increasing the likelihood of positive CMR findings. Outcome data remain outstanding. The imaging sequences used in this study have been well validated, standardized, and locked for the use in multicenter settings. The use of other imaging protocols, sequence parameters, or postprocessing approaches may yield different results.

## Conclusions

Taken together, we demonstrate cardiac involvement in 78 patients (78%) and ongoing myocardial inflammation in 60 patients (60%) with recent COVID-19 illness, independent of preexisting conditions, severity and overall course of the acute illness, and the time from the original diagnosis. These findings indicate the need for ongoing investigation of the long-term cardiovascular consequences of COVID-19.

## Notes

Supplement. **eFigure.** STROBE diagram
