# Supplementary material for: Outcomes of Cardiovascular Magnetic Resonance Imaging in Patients Recently Recovered From Coronavirus Disease 2019 (COVID-19)
Source: JAMA Cardiol. 2020 Jul 27;5(11):1265–73. doi: 10.1001/jamacardio.2020.3557 (PMC7385689; doi:10.1001/jamacardio.2020.3557)
Supplement: Supplement. — eFigure. STROBE diagram [file jamacardiol-e203557-s001.pdf]

Supplementary Online Content

Puntmann VO, Carerj ML, Wieters I, et al. Outcomes of cardiovascular magnetic resonance imaging in patients recently recovered from coronavirus disease 2019 (COVID-19). *JAMA Cardiol*. Published online July 27, 2020. doi:10.1001/jamacardio.2020.3557

eFigure. STROBE diagram

This supplementary material has been provided by the authors to give readers additional information about their work.

© 2020 Puntmann VO et al. *JAMA Cardiology.*

eFigure. STROBE diagram

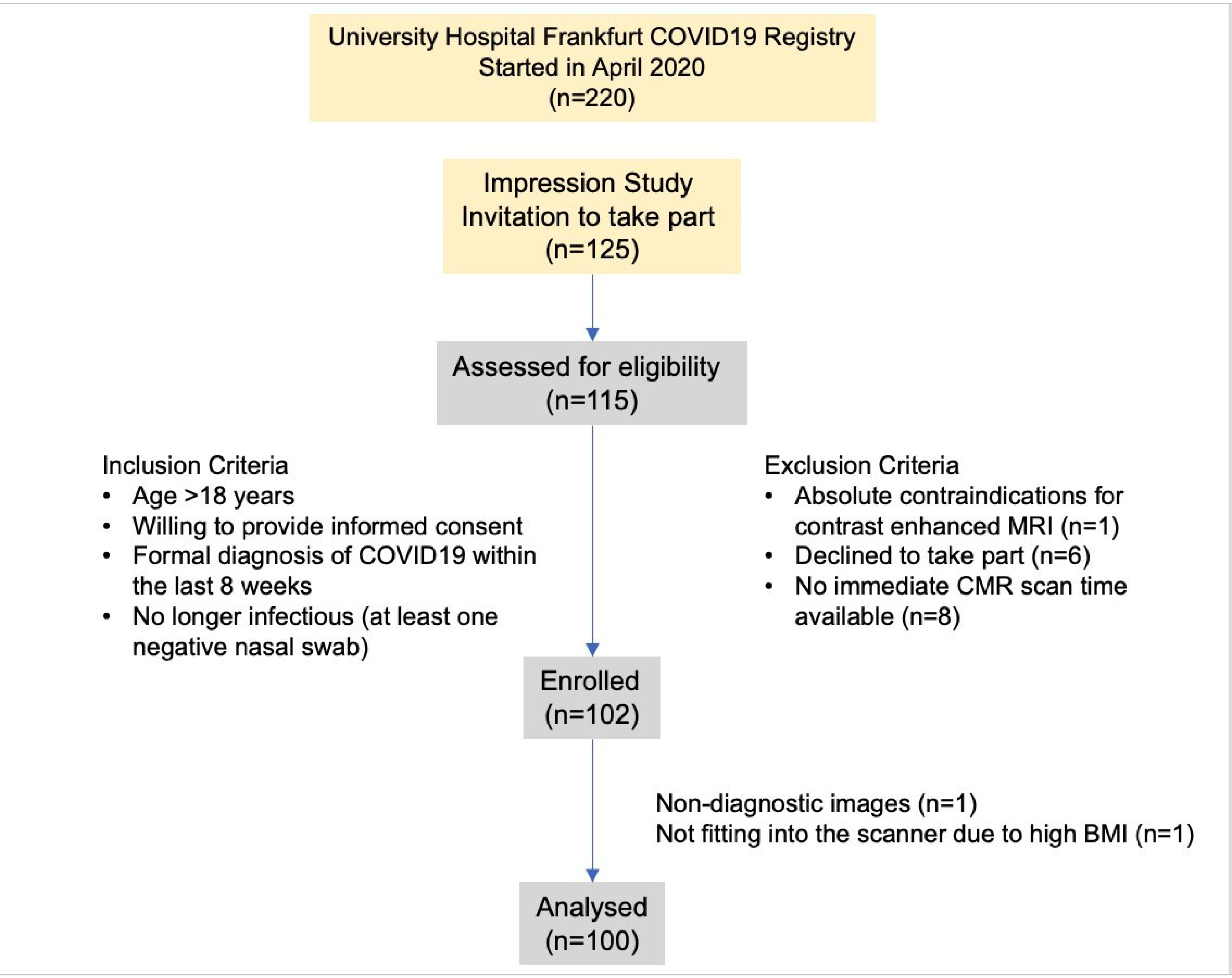

© 2020 Puntmann VO et al. *JAMA Cardiology.*
